# COVID-19 Bullous Lung Disease Superinfection by Raoultella planticola

**DOI:** 10.7759/cureus.39910

**Published:** 2023-06-03

**Authors:** Sriharsha Dadana, Raja Shekar Jadav, Anusha Kondapalli

**Affiliations:** 1 Internal Medicine, Cheyenne Regional Medical Center, Cheyenne, USA

**Keywords:** coronavirus disease 2019, raoultella planticola, covid-19, rare cause of infection, superinfection, bullous lung disease, raoultella

## Abstract

Bullous lung lesions from coronavirus disease 2019 (COVID-19) pneumonia, causing pneumothorax, are a rare complication, affecting up to 1% of infected patients. *Raoultella planticola* is an aerobic, gram-negative bacteria known to cause opportunistic infection. We present a rare case of spontaneous pneumothorax from rupture of lung bulla as a late sequela from COVID-19 pneumonia and superinfection of the bulla by *R. planticola*. Although superinfection of bullous lesions is known, this is the first reported case of *R. planticola* pneumonia in a patient with COVID-19 lung bullae. COVID-19 patients are at heightened risk for bullous lung lesions and superinfection by opportunistic organisms; thus, they should be followed up closely.

## Introduction

Cystic lung lesions from coronavirus disease 2019 (COVID-19) pneumonia, causing lung bullae and pneumothorax, are a rare complication, affecting up to 1% of infected patients [1,2]. *Raoultella planticola* is a gram-negative rod, aerobic, encapsulated bacteria of the *Enterobacteriaceae* family that is usually found in soil, plant, and aquatic environments [3]. It is a rare human pathogen known to cause opportunistic infections [4,5]. Previously reported cases of *R. planticola*-related infections include bacteremia, urinary tract infections, pneumonia, joint infections, and cholecystitis, among others [3,6-9]. In recent years, the number of *R. planticola*-related infections is on the rise, mainly nosocomial infections, and so is antibiotic resistance, as strains of multidrug-resistant carbapenemase-producing organisms are being reported more frequently [3,10-12]. We present a rare case of spontaneous pneumothorax and subcutaneous emphysema from rupture of lung bulla as a late sequela from COVID-19 pneumonia in a patient without needing positive pressure ventilation and superinfection of the bulla by *R. planticola*.

This case was presented as a thematic poster at the American Thoracic Society 2021 International Conference.

## Case presentation

**Figure 1:**
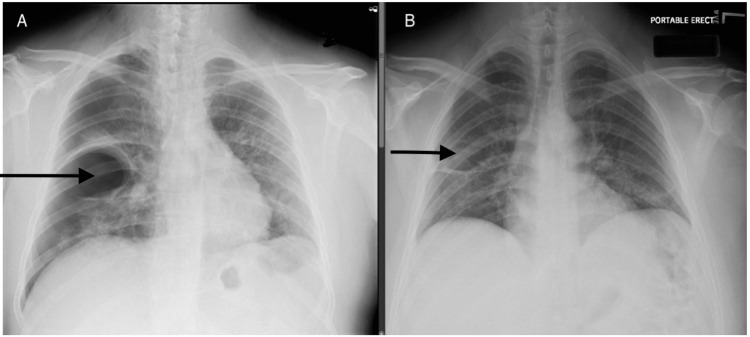
(A) Bullae in the right lung (arrow). (B) Near resolution of the right lung bullae (arrow)

**Figure 2:**
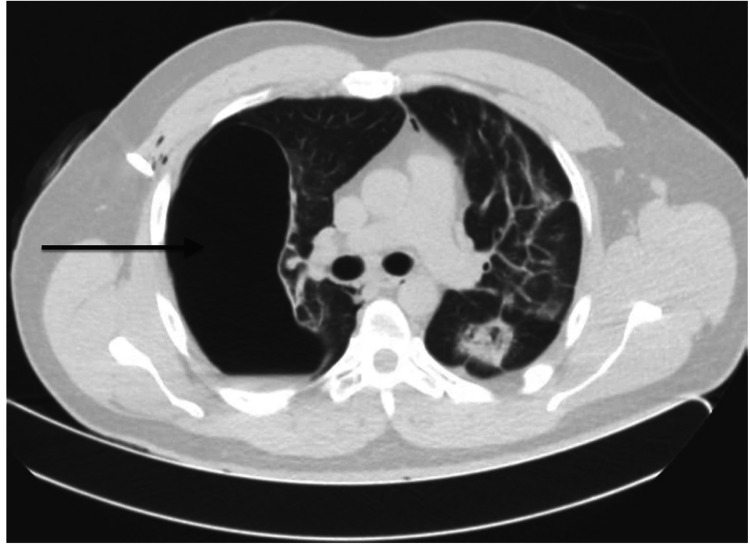
CT showing large right bullae (arrow)

A 37-year-old male presented to the emergency department (ED) with a sudden onset of sharp right-sided chest pain and shortness of breath after an episode of dry cough. He had no past medical history, worked in construction, and had never smoked. One month before the presentation, he tested positive for COVID-19 with mild symptoms of dry cough and lethargy, not requiring hospitalization. Vitals on arrival were as follows: blood pressure of 110/68 mmHg, heart rate of 92 beats/minute, respiratory rate of 26 breaths/minute, and oxygen saturation of 93% on room air. Physical exam was remarkable for mild respiratory distress with tachypnea. Electrocardiogram showed normal sinus rhythm. The chest radiograph (Figure 1) revealed right-sided pneumothorax and subcutaneous emphysema, with bleb formation at the right mid-lung field. He was given supplemental oxygen, and an emergent right-sided pigtail catheter tube was placed with marked interval improvement in aeration of the right hemithorax in a repeat chest radiograph. Polymerase chain reaction (PCR) from nasopharyngeal swabs confirmed COVID-19 infection. Serologic workup was remarkable for leukocytosis (17,000 cells/µL), elevated interleukin-6 level (22 pg/mL), erythrocyte sedimentation rate (ESR) at 34 mm/hour, and C-reactive protein (CRP) at 5.14 mg/dL. HIV p24 antigen and HIV-1/2 antibodies were not detected, and hepatitis B and C antibodies were negative. Computed tomography (CT) of the chest (Figure 2) showed minimal residual right pneumothorax, large right-sided bulla, bilateral patchy infiltrates containing air bronchograms, stellate borders, and visceral pleural extensions. Analysis of sputum was negative for bacteria and fungi, and PCR to detect *Mycobacterium tuberculosis* was negative. The serum eosinophil count was normal and was negative for the Aspergillus antigen. Further workup for antinuclear antibodies, anti-double-stranded DNA, anti-cardiolipin antibodies IgG/M, and anti-neutrophil cytoplasmic antibodies was negative. Subsequently, the patient's symptoms improved without any additional treatment, the chest tube was removed, and he was discharged to follow up in the outpatient clinic.

**Figure 3:**
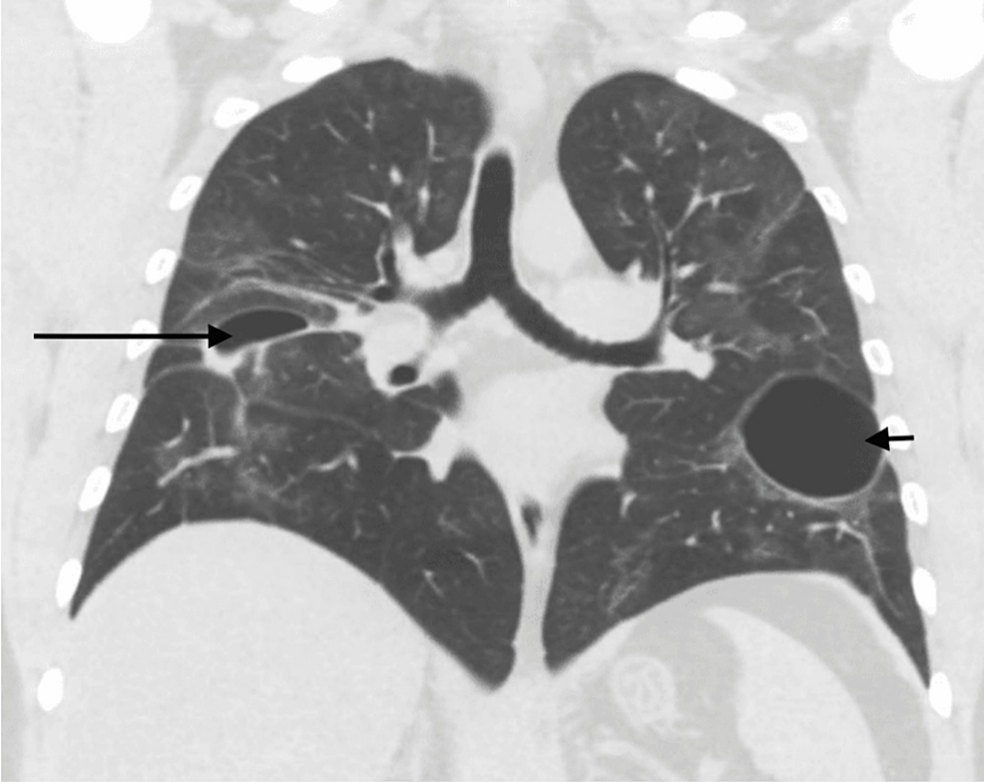
CT showing right lower lobe bulla (long arrow), which significantly decreased in size compared to the prior exam, with fluid and debris in the dependent portion and also showing new left lung bullae (short arrow)

During the post-discharge follow-up in the pulmonary clinic four weeks later, he complained of a cough with blood-tinged sputum, for which he was referred to the ED and subsequently was admitted. During this visit, he again tested positive for COVID-19 by PCR but without any symptoms except hemoptysis. The examination was unremarkable with no supplemental oxygen needed. Laboratory workup revealed leukocytosis at 11,000 cells/µL, ESR of 19 mm/hour, and normal CRP at 0.19 mg/dL. The chest radiograph (Figure 1B) showed linear opacities in the right mid-lung field without any evidence of an acute cardiopulmonary process. Repeat CT (Figure 3) revealed right lower lobe bulla, which significantly decreased in size compared to prior CT, with fluid and debris in the dependent portion, patchy ground-glass opacities throughout both lungs, and scattered bullae in the left lung, which were new from the prior exam (largest in left lower lobe measuring 5 x 4.5 cm axially and additional small bulla in left upper and lower lobes measuring up to 1.3 to 2 cm, respectively). CT scan also revealed fluid and debris in the dependent portion of bullae that led to suspected pneumonia due to bullae superinfection. Sputum culture was positive for *R. planticola*, pan-sensitive, and the patient was started on amoxicillin-clavulanic acid with improvement in symptoms, and subsequently discharged on seven days course of antibiotics.

In the pulmonary clinic follow-up after a month, the patient was asymptomatic, and a repeat third CT of the chest showed resolved bullae with scarring.

## Discussion

Bullous lung disease, pneumatocele, and pneumothorax are rare complications of COVID-19 infection [13]. Mechanical ventilation is known to be a major risk factor for pneumothorax in COVID-19 pneumonia, although it can occur in patients who did not require mechanical ventilation [2,14,15]. The release of cytokines causing inflammatory damage to the alveoli and airway, resulting in the weakening of bronchial walls with prolonged coughing is a possible trigger [2,14]. Although the superinfection of bullous lung lesions is known, this is one of the rare cases of *R. planticola* pneumonia caused by the superinfection of COVID-19 lung bullae. The only symptom of COVID-19 infection in this patient was a dry cough; new bullae were seen at different time periods (one month and two months) after the initial infection. Superinfection by *R. planticola* causing pneumonia likely led to the blood-tinged sputum, and this patient’s occupation as a construction worker might be the source of infection from the soil. This case will help us understand that bullous lung lesions may occur months after infection by COVID-19, even in patients with mild symptoms, irrespective of mechanical ventilation status, and these patients are at heightened risk for pneumothorax and superinfection by opportunistic organisms; thus, these patients should be followed up closely. CT scan of the chest is the mainstay to diagnose such pathologies and determine disease severity with or without co-infection with other organisms [1].

*R. planticola* inhabits natural environments like water, soil, and plants [3]. A total of 9-18% of humans are colonized with this bacterium in the gastrointestinal and upper respiratory tract as their reservoirs [10,16]. It is a gram-negative, aerobic, rod-shaped bacterium belonging to the *Enterobacteriaceae* family, pathogenetically similar to *Klebsiella* spp. [16]. It is known to cause infections in cases of trauma with soil contamination, nosocomial infections in patients who underwent invasive procedures, and immunocompromised states enabling the dormant colonizers to become invasive [3,16]. The first case of community-acquired pneumonia with *R. planticola* has been reported in Korea [17]. Previously, it is not typically known to cause severe infections in humans, though, in recent years, it is known to be associated with severe nosocomial infections in immunocompromised as well as immunocompetent patients [7,16,18-20]. While most of the *R. planticola* isolates are sensitive to a wide range of antibiotics, including cephalosporins and carbapenems, the number of reported cases of carbapenem-resistant strains of *R. planticola* (via the production of carbapenemases) are on the rise, mainly causing hospital-acquired pneumonia [10-12]. The emergence of carbapenem-resistant blaIMP-8 strains of *Raoultella* was first studied in 2014 by Tseng et al. [11]. Although only a few cases of mortalities have been reported associated with *R. planticola* infection relating to its carbapenemase-producing abilities, physicians should be vigilant about their potential for the emergence of multidrug-resistant strains.

## Conclusions

To our knowledge, this is one of the few cases of *R. planticola* superinfection causing pneumonia in a patient with COVID-19 bullous lung disease. Patients infected with COVID-19 are at heightened risk for bullous lung disease, irrespective of their mechanical ventilation status and superinfection by opportunistic organisms. Thus, these patients should be followed up closely, and there should be increased awareness about *R. planticola* and other opportunistic infections, its emerging resistance pattern, and the potential for nosocomial outbreaks.
